# Emerging novel agents for patients with advanced Ewing sarcoma: a report from the Children’s Oncology Group (COG) New Agents for Ewing Sarcoma Task Force

**DOI:** 10.12688/f1000research.18139.1

**Published:** 2019-04-15

**Authors:** Kelly Bailey, Carrye Cost, Ian Davis, Julia Glade-Bender, Patrick Grohar, Peter Houghton, Michael Isakoff, Elizabeth Stewart, Nadia Laack, Jason Yustein, Damon Reed, Katherine Janeway, Richard Gorlick, Stephen Lessnick, Steven DuBois, Pooja Hingorani

**Affiliations:** 1 Division of Pediatric Hematology/Oncology, University of Pittsburgh School of Medicine, Pittsburgh, PA, USA; 2 Center for Cancer and Blood Disorders, Department of Pediatrics, University of Colorado School of Medicine, Aurora, CO, USA; 3 Departments of Pediatrics and Genetics, Lineberger Comprehensive Cancer Center, University of North Carolina, Chapel Hill, NC, USA; 4 Department of Pediatrics, Memorial Sloan-Kettering Cancer Center, New York, NY, USA; 5 Departement of Pediatrics, Van Andel Institute, Helen De Vos Children’s Hospital and Michigan State University, Grand Rapids, MI, USA; 6 Greehey Children’s Cancer Research Institute, University of Texas Health Science Center, San Antonio, TX, USA; 7 Center for Cancer and Blood Disorders, Connecticut Children’s Medical Center, Hartford, CT, USA; 8 Department of Oncology, St. Jude Children’s Research Hospital, Memphis, TN, USA; 9 Department of Radiation Oncology, Mayo Clinic, Rochester, MN, USA; 10 The Faris D. Virani Ewing Sarcoma Center at the Texas Children’s Cancer Center, Baylor College of Medicine, Houston, TX, USA; 11 AYA Program, Moffitt Cancer Center, Tampa, FL, USA; 12 Johns Hopkins All Children’s Hospital, St. Petersburg, FL, USA; 13 Dana-Farber/Boston Children’s Cancer and Blood Disorders Center and Harvard Medical School, Boston, MA, USA; 14 Division of Pediatrics, University of Texas MD Anderson Cancer Center, Houston, TX, USA; 15 Center for Childhood Cancer and Blood Diseases, Research Institute at Nationwide Children’s Hospital, Columbus, OH, USA; 16 Division of Pediatric Hematology/Oncology/Bone Marrow Transplantation, The Ohio State University College of Medicine, Columbus, OH, USA; 17 Center for Cancer and Blood Disorders, Phoenix Children's Hospital, Phoenix, AZ, USA

**Keywords:** Ewing sarcoma, metastasis, relapse, clinical trials, therapy

## Abstract

Ewing sarcoma is a small round blue cell malignancy arising from bone or soft tissue and most commonly affects adolescents and young adults. Metastatic and relapsed Ewing sarcoma have poor outcomes and recurrences remain common. Owing to the poor outcomes associated with advanced disease and the need for a clear research strategy, the Children’s Oncology Group Bone Tumor Committee formed the New Agents for Ewing Sarcoma Task Force to bring together experts in the field to evaluate and prioritize new agents for incorporation into clinical trials. This group’s mission was to evaluate scientific and clinical challenges in moving new agents forward and to recommend agents and trial designs to the Bone Tumor Committee. The task force generated a framework for vetting prospective agents that included critical evaluation of each drug by using both clinical and non-clinical parameters. Representative appraisal of agents of highest priority, including eribulin, dinutuximab, cyclin-dependent kinase 4 and 6 (CDK4/6) inhibitors, anti-angiogenic tyrosine kinase inhibitors, and poly-ADP-ribose polymerase (PARP) inhibitors, is described. The task force continues to analyze new compounds by using the paradigm established.

## Introduction

Ewing sarcoma (ES) is a small round blue cell bone tumor most commonly occurring in adolescent and young adult (AYA) patients. ES is a classic fusion oncoprotein-driven tumor typically associated with a reciprocal translocation involving *EWSR1* (chromosome 22) and the ETS family transcription factor *FLI1* (chromosome 11) ^1^. These fusions may arise from chromoplexy events ^2^. Whereas classically no known genetic predisposition syndromes were linked to ES tumor development, recent evidence suggests that germline mutations in genes regulating DNA damage pathways are associated with an increased risk of developing ES ^3^. The field continues to learn more about the origins, nature, and behavior of the EWS-FLI1 oncoprotein in the context of individual tumor cells and the micro-environment ^4, 5^. This knowledge could assist in the future discovery of new therapeutic targets and in developing guidelines for risk stratification and treatment.

Currently, the strongest prognostic factor is stage at initial diagnosis. In North America, survival in localized ES improved to more than 70% 5-year event-free survival (EFS) by treating with alternating cycles of interval-compressed vincristine, doxorubicin, cyclophosphamide and ifosfamide and etoposide (VDC/IE) ^6^. The Euro-Ewing 99 clinical trial improved outcomes in localized ES with high-risk features by intensification of therapy with high-dose chemotherapy with busulfan and melphalan ^7^. The addition of IE cycles in earlier studies or intensification of therapy with high-dose chemotherapy has not improved outcomes for patients with metastatic disease ^8, 9^. As patients with metastatic ES have not benefited from treatment intensification, it suggests that targeted agents will be needed for this population. As an example, an ongoing Children’s Oncology Group (COG) trial is evaluating the insulin-like growth factor 1 receptor (IGF-1R) antibody ganitumab with cytotoxic chemotherapy (ClinicalTrials.gov Identifier: NCT02306161) ^10^. Patients with relapsed ES also have a dismal prognosis, and survival estimates are about 10% ^1^. Current efforts in the relapsed setting have focused on targeting EWS-FLI1 itself, targeting DNA damage vulnerabilities, or exploring immunotherapy strategies ^11– 20^. For the purpose of this report, we consider patients with either newly diagnosed metastatic or relapsed ES to have advanced disease. There is a dire need for new therapies to improve outcomes for patients with advanced ES.

It is in this clinical climate that the COG Bone Tumor Committee established the New Agents for Ewing Sarcoma Task Force. The COG Bone Tumor Committee previously established a successful working group for drug development in osteosarcoma ^21^. The purpose of this ongoing effort in ES is to bring together experts (basic scientists, experts in preclinical testing, pediatric sarcoma clinicians, and clinical investigators) with the primary goal of identifying potential agents of high priority for clinical evaluation and expeditiously incorporating these agents into clinical trials. Many new agents are being investigated in relapsed ES and many of these are listed in Table 1. In this report, we summarize our work to date, including establishing a framework to prioritize potential agents in this rare disease, characterizing challenges in understanding the disease biology, determining the bar for preclinical data, recommending an appropriate cytotoxic chemotherapy backbone on which to layer novel agents, and highlighting practical considerations for clinical trial development in the advanced ES patient population. We aim to provide clinicians and basic scientist’s insight into our approach in order to broadly facilitate discussions on moving new agents forward in this rare tumor. For additional background on ES more generally, we refer the reader to a recent review article ^22^.

**Table 1.** Agents discussed and evaluated by New Agents for Ewing Sarcoma Task Force in 2018. The agents appearing in a bold typeface are expanded upon in Table 2.

Drug/Target Class	Example drugs
EWSR1-FLI1 Target agents: Splicing inhibitors Minor groove-binding agents	TK-216 Mithramycin, Trabectedin and Lurbinectidin
Epigenetic therapies	Lysine-specific demethylase 1A (LSD1) inhibitors (seclidemstat and IMG-7289) Histone deacetylase inhibitors (vorinostat, entinostat, and panobinostat) Bromodomain inhibitors
CD99 targeting agents	Clofarabine/Cladribine and CD99 antibody
Novel cytotoxic agents	**Eribulin**, aldoxorubicin and palifosfamide
Multi-targeted tyrosine Kinase Inhibitors	**Pazopanib, regorafenib** and **cabozantinib**
Mammalian target of rapamycin (mTOR) inhibitors	**Nab-Rapamycin**, temsirolimus
DNA damage/Repair	Poly-ADP-ribose polymerase (PARP) inhibitors **(niraparib, olaparib, talazoparib)** Wee1 inhibitors (AZD1775) CHK1 inhibitors (prexasertib)
Cell cycle cyclin-dependent kinase (CDK) inhibitors	CDK4/6 Inhibitors **(palbociclib, ribociclib, abemaciclib)**
Transcriptional CDK Inhibitors	CDK7 inhibitor (SY-1365) CDK12 inhibitor
MDM2 Inhibitor	AMG-232, DS-3032b, ALRN-6924 and idasanutlin
Insulin-like growth factor 1 receptor (IGF-1R) inhibitors	**Ganitumab**
Platelet-derived growth factor receptor (PDGFR) antibodies	Olaratumab
Other monoclonal antibodies	MORab-004
Metabolic modulators	Nicotinamide phosphoribosyltransferase (NAMPT) inhibitors and Metformin
Immunotherapy	GD2 antibody **(dinutuximab)** VIGIL/FANG Chimeric antigen receptor (CAR) T cells

## Conceptual framework for assessing novel agents in advanced Ewing sarcoma

**Figure 1.**
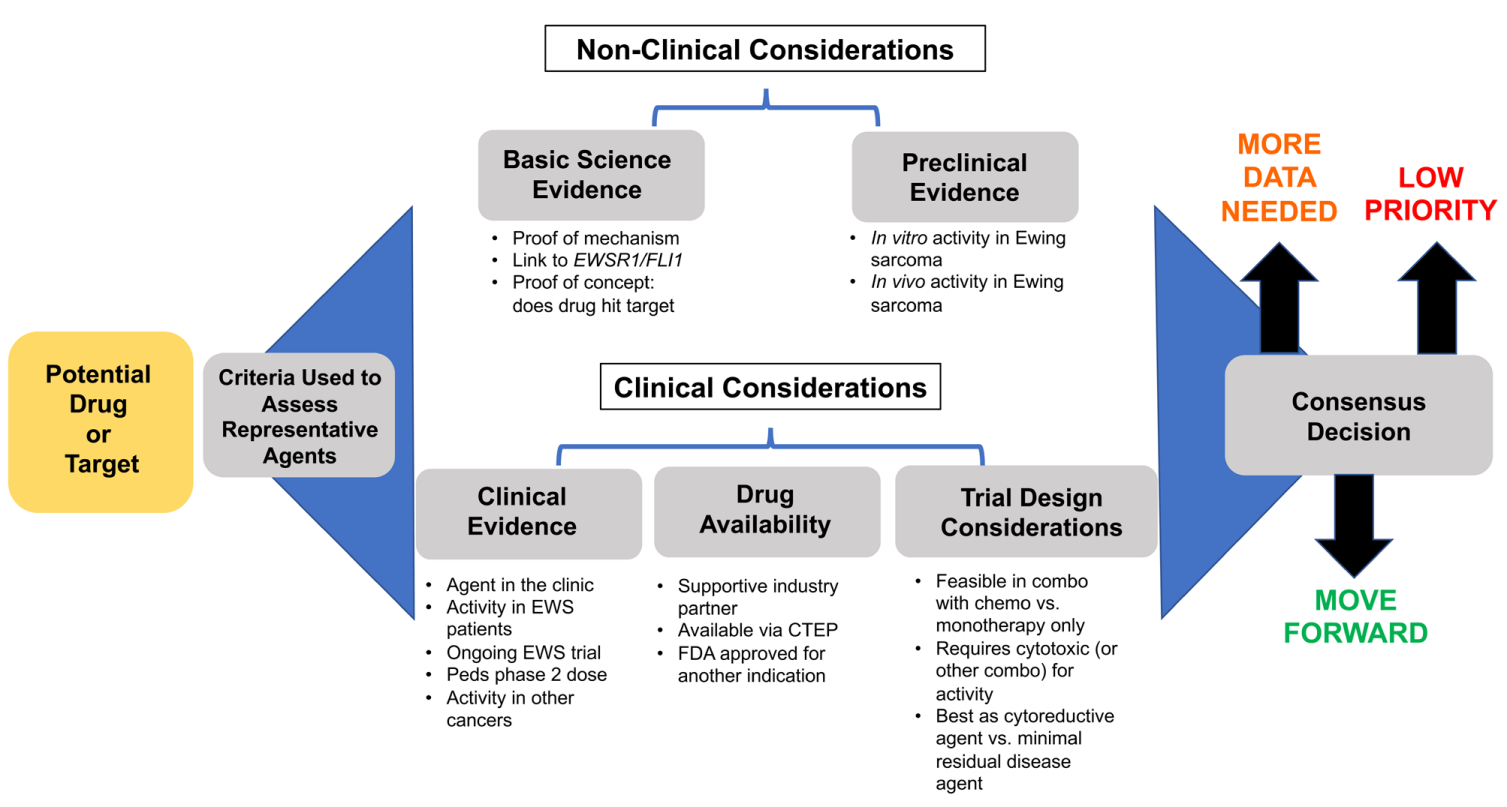
Paradigm for evaluation new agents for Ewing sarcoma. Task force members proposed agents or targets (detailed in Table 1). These proposals were then each individually discussed using the step-wise approach outlined. Agents deemed worthy to move forward were then re-examined and re-vetted through this work flow as new preclinical or trial data updates became available. CTEP, Cancer Therapy Evaluation Program; EWS, Ewing sarcoma; FDA, US Food and Drug Administration.

The task force defined key criteria against which to gauge potential new agents of relevance to ES ( Figure 1). These criteria were broadly divided into non-clinical and clinical criteria.

### Non-clinical criteria

The fundamental non-clinical criterion is the strength of the basic science evidence demonstrating that the target either is critical to ES pathogenesis or has an expression pattern that is relatively specific to ES. Mechanistic dependency may be linked directly to targeting the oncoprotein EWS-FLI or effectors downstream of EWS-FLI1 or micro-environmental factors critical to the tumor. Once a target is identified, it is crucial to have proof-of-concept data that a putative drug is active against the intended target.

In addition to proof-of-concept data, the remaining requirements for preclinical data will likely vary greatly depending on the agent and the model systems available. For example, although ideally animal model data would be obtained in the preclinical setting, acquiring animal data for immunotherapy agents is currently not optimal given the lack of transgenic and well-developed humanized mouse models of ES ^23^. Although a range of *in vitro* and *in vivo* supportive preclinical testing can be considered to help prioritize agents for further development, for a rare tumor such as ES for which relapse is nearly uniformly fatal, extensive preclinical testing need not be required if the rationale for the agent is otherwise strong. Preclinical data in ES have not always predicted clinical response; furthermore, the degree of testing is not standardized and particular attention to pharmacokinetics, pharmacodynamics, and the use of a clinically relevant dose and schedule is recommended to provide translational relevance ^24– 28^.

### Clinical criteria

From a clinical perspective, the paramount criterion is a signal of activity in early-phase testing with agents meeting this criterion prioritized for rapid translation into a trial for relapsed or newly diagnosed metastatic populations. Drug availability, through US Food and Drug Administration (FDA) approval for other indications, inclusion in the Cancer Therapy Evaluation Program (CTEP) portfolio, or collaboration with an engaged industry partner, offers another factor in prioritization. Availability of pediatric dosing schedules is an advantage but not a prerequisite in this AYA cancer ^29– 31^. Given that single-agent therapy is unlikely to be curative in most circumstances, it is important to consider the feasibility of combination therapy. Other clinical parameters discussed are outlined in Figure 1.

With these non-clinical and clinical criteria in place, we formulated a list of potential agents for advanced ES. Table 1 is an inclusive list of agents considered in 2018. We next methodically considered each agent in a step-wise fashion, applying the aforementioned criteria. We applied these criteria to selected potential agents of greatest interest ( Table 2). Table 2 also provides a benchmark for ganitumab, an agent currently in phase 3 testing in newly diagnosed metastatic ES, and for mammalian target of rapamycin (mTOR) inhibitors currently proposed for the next COG trial for first recurrence.

**Table 2.** Work-flow summary of the top five promising agents in 2018. Work-flow summary of eribulin, dinutuximab, tyrosine kinase inhibitors, cyclin-dependent kinase 4 and 6 (CDK4/6) inhibitors, and poly-ADP-ribase polymerase (PARP) inhibitors. Ganitumab (currently in a metastatic Ewing sarcoma clinical trial) and mammalian target of rapamycin (mTOR) inhibitors (currently in a trial proposal) are also included using the task force’s work flow for comparison.

Drug	Basic Science Evidence	Preclinical Evidence	Clinical Evidence	Drug Availability	Trial Design Consideration	Consensus Decision
**Ganitumab** (in trial)	++	+	++	+	++	In trial (AEWS1221)
**mTOR inhibitor** **(proposed trial)**	++	+	+	++	++	Move forward
**Anti-GD2** **monoclonal**	++		+	++	++	Move forward
**Eribulin**	+	+	+	++	++	In early trials
**Multi-targeted** **tyrosine kinase** **inhibitor**	+	+	+	++	+	In early trials
**CDK4/6 inhibitor**	++	++		++	+	In early trials
**PARP inhibitor**	++	++	-	++	-	In early trials

**Table 2 - = negative data, + =some evidence, ++ =significant evidence, blank=no data**

## Eribulin

Eribulin is a microtubule inhibitor that inhibits polymerization of tubulin subunits and differs from other microtubule inhibitors by preventing lengthening and shortening of microtubules during division. Aggregation of unstable tubulin polymers ultimately results in cellular apoptosis ^32– 35^. EWS-FLI1 is known to drive the expression of proteins that regulate microtubule stability ^15^. Preclinical testing of ES cell lines demonstrated apoptosis, induced through the Bcl-2 pathway ^36^. Eribulin was also evaluated by the Pediatric Preclinical Testing Program (PPTP); four out of five ES xenografts demonstrated a complete response to treatment ^37^. In sarcoma treatment, eribulin is FDA-approved for adult patients with liposarcoma who previously received an anthracycline ^38^. The COG performed a phase 1 trial of eribulin in children with advanced solid tumors; one of the evaluable patients with ES experienced a partial response for four cycles ^39^. An ongoing phase 2 trial (ClinicalTrials.gov Identifier: NCT03441360) is assessing eribulin in patients with relapsed ES ^40^. An ongoing phase 1/2 trial (ClinicalTrials.gov Identifier: NCT03245450) is evaluating eribulin in combination with irinotecan, although these agents are not expected to have significant overlapping toxicities ^41^. Furthermore, vincristine and topoisomerase 1 inhibitors act synergistically in xenograft models and in pediatric clinical trials; therefore, eribulin with irinotecan may also have clinical synergy ^42, 43^.

Eribulin shows strong preclinical data in ES, is an FDA-approved agent for another sarcoma indication, and has led to at least one monotherapy response in relapsed ES. We will recommend further trial development with eribulin as a component of combination therapy pending the results of current trials.

## Dinutuximab

GD2 is a disialoganglioside that is expressed on the surface of tumors of neural crest origin, such as neuroblastoma ^44^. The precise cell of origin for ES is unknown; however, it is thought to be a neural crest or mesenchymal-derived tumor ^45– 47^. ES tumors have been evaluated for GD2 expression, and results range from no detectable surface expression to diffuse/intense staining in some tumors ^48– 51^. It is not known whether GD2 expression changes upon relapse or whether levels differ in patients with newly diagnosed metastatic ES. Several available antibodies in the clinic are known to bind to this target, including dinutuximab, which is approved by the FDA for use in neuroblastoma. There is currently no preclinical evidence for utility of dinutuximab in ES.

The clinical evidence supporting dinutuximab borrows heavily from another GD2-positive pediatric cancer, neuroblastoma. The combination of irinotecan, temozolomide, and dinutuximab has been shown to demonstrate significant clinical benefit in children with advanced neuroblastoma ^52^. Pediatric dosing, schedules, and toxicities are well documented with this therapy combination. Patients with relapsed neuroblastoma are generally younger than patients with relapsed ES, and there is the potential for different toxicity in older patients. As some ES tumors express GD2, determining whether the addition of dinutuximab to chemotherapy extends to ES is a logical clinical trial question. Dinutuximab is FDA-approved and also available in the CTEP portfolio. As demonstrated in neuroblastoma trials, dinutuximab is both feasible and more effective in combination with irinotecan and temozolomide (IT).

The task force determined that the available clinical data in another GD2-positive tumor treated with this combination are sufficiently strong to nominate this approach for clinical evaluation in ES. Irinotecan and temozolomide are agents routinely used in relapsed ES therapy, and a next-step trial could determine the efficacy of dinutuximab added to IT. The combination would be strategically combining immune-based treatment with traditional cytotoxic therapy. Given the current inconsistency in GD2 immunohistochemistry staining, it would not be possible to select patients upfront on the basis of tumor GD2 expression.

## Anti-angiogenic tyrosine kinase inhibitors

A large body of work highlights the role of angiogenesis in ES ^53– 56^. Several tyrosine kinase inhibitors (TKIs) targeting angiogenesis have been developed, although most are multi-targeted TKIs that target other receptor tyrosine kinases (RTKs). RTKs are important regulators of cell growth, proliferation, and survival. Aberrant RTK signaling resulting from amplification, mutation, or overexpression has been implicated in many cancers, including ES ^57^.

Pazopanib primarily targets VEGFR-1 and -2, PDGFR-α and -β, and c-Kit ^58^. Pazopanib has gained FDA approval for treatment of refractory soft tissue sarcoma (STS) in adult patients. A phase 3 clinical trial for patients with advanced STS demonstrated improved progression-free survival (PFS) (4.6 months) in the pazopanib arm compared with placebo (1.6 months) ^59^. The PPTP evaluation of pazopanib revealed a statistically prolonged EFS in ES xenografts but no objective responses ^60^. Several case reports have demonstrated partial responses in patients with ES; however, resistance seems to develop with prolonged use ^61– 63^. Cabozantinib targets VEGFR2, c-MET, and AXL and is FDA-approved for the treatment of medullary thyroid cancer and renal cell carcinoma in adults. A phase 2 study of cabozantinib in patients with recurrent ES showed tumor control with 9 (27.7%) partial responses and 10 (30.3%) with stable disease ^64^. Pediatric phase 2 dosing is established for both pazopanib and cabozantinib ^65, 66^. Regorafenib is an oral multikinase inhibitor that targets VEGFR-1-3, FGFR1, PDGFR-α and -β, CSFR-1, and c-Kit. It has FDA approval for use in metastatic colorectal carcinoma, gastrointestinal stromal tumor, and hepatocellular carcinoma. SARC024 evaluated regorafenib in 30 patients with ES. The median PFS was 3.6 months, and the median duration of response was 5.5 months ^67^.

At least three anti-angiogenic TKIs have shown clinical monotherapy activity in relapsed ES. This clinical activity together with preclinical rationale and drug availability should motivate evaluation of this class of agents in combination with cytotoxic chemotherapy or other targeted agents or as a potential maintenance therapy in future trials.

## CDK4/6 inhibitors

*CDKN2A* is a gene that codes for two proteins: p16 and p14arf. Both proteins are tumor suppressors; p16 inhibits cyclin-dependent kinases 4 and 6 (CDK4 and CDK6) by phosphorylating the RB protein, thus preventing cell cycle progression ^68^. About 13 to 30% of ES tumors have deletions in *CDKN2A* ^69– 71^. *CDKN2A* deletion does not appear to be associated with clinical outcome ^72^. Owing to the alteration of p16 in a subset of ES, there is clinical interest in CDK4/6 inhibitors that target and inhibit this pathway. The cyclin D1 gene has been shown to be associated with a superenhancer in ES ^73^. ES cells consequently have an activated cyclin D1/CDK4 pathway and require CDK4 and cyclin D1 for growth. *In vivo* data demonstrated decreased tumor growth and prolonged survival with CDK4/6 inhibition ^73^. These data provide a rationale for use even in the absence of *CDKN2A* deletions ^74^.

Currently, three CDK4/6 inhibitors have been FDA-approved for advanced breast cancer: abemaciclib, palbociclib, and ribociclib. These agents each have ongoing or completed single-agent pediatric phase 1 trials (ClinicalTrials.gov Identifiers: NCT02644460, NCT01747876, and NCT02255461) ^75– 77^. As in adults, the predominant toxicity seen in children is hematologic ^78^. Since single-agent therapy is rarely curative in sarcomas, there is little interest in evaluating single-agent CDK4/6 inhibitors in patients with ES. Combination with conventional cytotoxic chemotherapy agents, many of which are dependent on S-phase cycling, may be challenging because of antagonistic mechanisms ^79^. Furthermore, the significant myelosuppression seen with CDK4/6 inhibitors complicates combinatorial therapy. Without significant adult data evaluating CDK4/6 inhibitors with chemotherapy, we await the results of a planned trial evaluating palbociclib with IT before recommending a chemotherapy combination approach in advanced ES (ClinicalTrials.gov Identifier: NCT03709680) ^80^. There are other trials in the US combining CDK4/6 inhibitors with agents, including MEK and mTOR inhibitors (ClinicalTrials.gov Identifiers: NCT03387020, NCT03114527, and NCT02703571) ^81– 83^. Other preclinical data suggest that dual inhibition of CDK4/6 and IGF-1R may be a combination to consider in ES, as CDK4/6 drug resistance is mediated by activation of IGF-1R signaling ^84^. In sum, CDK4/6 inhibitors have potential efficacy in ES but identifying the appropriate combination therapy has been challenging. We recommend additional preclinical and clinical data before moving this class of drugs forward in first-relapse or metastatic ES.

## PARP inhibitors

Much enthusiasm has surrounded poly-ADP-ribose polymerase 1 (PARP1) inhibitors in ES. PARP plays a significant role in DNA repair, particularly with single-strand DNA damage. Inhibition of PARP proteins can cause persistent single-strand breaks, ultimately resulting in cellular apoptosis. EWS-FLI1 interacts with PARP1, influencing its transcriptional activity, and ES tumors have high levels of PARP mRNA and protein activity ^85^. Several PARP inhibitors, including olaparib, rucaparib, talazoparib, and niraparib, have received FDA approval for the treatment of ovarian or breast cancer in adult patients. Interest in PARP inhibitors in ES resulted from preclinical data demonstrating sensitivity in ES cell lines ^86^. A phase 2 trial with olaparib monotherapy quickly opened for adults with relapsed ES. Of the 12 patients enrolled, none had objective responses and four patients had stable disease. The median time to progression was 5.7 weeks ^25^. The PPTP and others evaluated PARP inhibitors in combination with DNA-damaging chemotherapy and demonstrated activity in ES xenografts ^87– 89^.

Owing to these promising preclinical data, a series of successor trials have evaluated PARP inhibitors in combination with irinotecan, temozolomide, or IT (ClinicalTrials.gov Identifiers: NCT02116777, NCT01858168, NCT02044120, and NCT02392793) ^90– 93^. While there have been hints of clinical activity, preliminary presentations of the toxicity data have shown that the myelosuppression of PARP inhibitors with cytotoxic chemotherapy is limiting dose intensity in these combination trials ^94^. This drug class demonstrates that strong preclinical activity and rationale may not always predict clinical feasibility, particularly in a heavily pretreated, relapsed patient population. At this time, we recommend awaiting results from ongoing clinical trials prior to moving this class of drugs further in advanced ES clinical trials.

## The cytotoxic backbone: trial recommendations

We also discussed the chemotherapy backbone onto which selected agents could be added for patients with first recurrent ES. Currently, there is no established standard backbone for patients with recurrent or refractory ES. Topotecan with cyclophosphamide has shown activity in patients with relapsed ES with response rates between 23 and 41% ^95– 100^. Owing to this promising activity, the COG conducted a pilot trial adding vincristine, topotecan, and cyclophosphamide (VTc) to interval-compressed ES therapy. This combination was tolerable, but hematologic toxicity was between 44 and 63% ^101^. The Euro Ewing Consortium is evaluating topotecan and cyclophosphamide in the rEECur trial (EudraCT number: 2014-000259-99) for recurrent ES. A randomized COG phase 3 trial, AEWS1031, evaluated the efficacy of adding VTc to the interval-compressed five-drug backbone, and results are pending. Owing to the significant hematologic toxicity of this combination, incorporating additional agents to this backbone may prove challenging but could be considered for the appropriate novel agents.

The combination of vincristine and IT (VIT) has also shown activity in patients with relapsed ES. VIT has demonstrated response rates between 29 and 63% in relapsed or refractory patients, although the actual VIT response rate has not yet been investigated in a prospective randomized trial ^102– 105^. This chemotherapy has schedule-dependent synergy ^106, 107^. The combination of drugs also has limited overlapping toxicity. Diarrhea and abdominal pain are the most common dose-limiting toxicities of irinotecan ^108, 109^. The major dose-limiting toxicity of temozolomide is myelosuppression ^110– 112^. Currently, the Euro Ewing Consortium is evaluating IT in relapsed ES. IT has a strong history of efficacious combination with monoclonal antibodies or targeted therapies in other diseases ^52, 113, 114^. Currently, there are ongoing trials for patients with ES combining IT with PARP inhibitors or Vigil autologous vaccine (ClinicalTrials.gov Identifiers: NCT02044120, NCT01858168, and NCT03495921) ^90, 92, 93, 115^. Owing to the reported efficacy of IT in ES and successful combination with other novel therapeutics, we believe IT (with or without vincristine) may be a preferred and feasible cytotoxic backbone for future combination clinical trials in first recurrent ES.

Although the original addition of IE to VDC did show improved outcomes for localized disease, patients with upfront metastatic ES saw no clinical benefit from this addition ^8, 116, 117^. Given these data, the group also discussed eliminating or reducing IE cycles for metastatic patients to decrease toxicity, allowing the addition of other targeted agents to VDC in future trials. Furthermore, early data from a single-institution trial including patients with upfront metastatic ES (ClinicalTrials.gov Identifier: NCT01864109) demonstrate the feasibility of incorporating IT into standard upfront therapy for metastatic ES ^118^. Altering the number of IE cycles and adding IT to the upfront chemotherapy backbone are both considerations for further trial design considering drug synergism and overlapping toxicities of the targeted agents being studied.

## Additional key clinical trial design considerations

In addition to establishing recommendations for advanced ES cytotoxic backbones, we examined other important aspects of trial design including patient randomization, maintenance therapy, and specific differences in trial design approach for patients with upfront metastatic versus relapsed ES. Given the rarity of this tumor, we continue to support collaborative, multi-national trials in order to study this cancer in a timely and inclusive manner.

With a goal of testing novel agents in patients with advanced ES, we propose several potential trial designs depending upon the agent and specific population of interest. The newly diagnosed metastatic population presents two potential opportunities for evaluating new agents. For agents expected to combine well with conventional cytotoxic chemotherapy, we recommend a randomized design comparing chemotherapy alone versus chemotherapy plus a novel agent. Depending upon the available evidence supporting the agent, the statistical parameters for such a trial may incorporate a phase 2 design with early stopping rules or a phase 3 trial to show definitive efficacy. For agents difficult to combine with chemotherapy, those that may be administered on a chronic schedule, or those with expected efficacy in minimal residual disease settings, a maintenance design could be considered. In this design, patients in a radiographic complete remission would continue treatment with the novel agent upon completion of frontline therapy. Both approaches are used in the ongoing COG trial of ganitumab. Given the paucity of clinical data for maintenance strategies in ES, this approach is best studied in the context of a randomized study, comparing maintenance with no maintenance or comparing two different promising maintenance strategies. Likewise, the type of maintenance regimen should be carefully considered in light of concerns about toxicity and adherence in an AYA population ^119^.

For the relapse population, there are two main design considerations. Given that ES often remains chemotherapy-responsive at first relapse, trial designs should incorporate cytotoxic chemotherapy. Therefore, testing of new agents can be performed as a single-arm trial combining a new agent with a standard backbone, and inference about activity of the combination is based on strong historical data for the backbone regimen. Alternatively, new agents may be tested in a randomized manner, either as a definitive comparison against the backbone alone or in a selection design comparing two novel agents added to the same backbone. Promising agents that are unlikely to pair well with chemotherapy (for example, antagonism; overlapping toxicity) may be best evaluated as monotherapy in a second relapse population.

## Conclusions and future directions

We have generated a robust group and infrastructure for vetting novel agents for evaluation in patients with ES. Future efforts by our group and other groups may use this paradigm as new agents or concepts become available. We discussed several strategies that were determined to be too early in development for immediate translation into a clinical trial for the newly diagnosed metastatic or first recurrent populations. Examples of these strategies include lysine-specific demethylase 1A (LSD1) inhibition, inhibition of interaction between EWSR1-FLI1 protein with RNA helicase, epigenetic modification of chromatin modeling resulting in EWS-FLI1 suppression, inhibition of transcriptionally active CDKs, and immunotherapy approaches in ES ^17, 120– 122^. The first two of these approaches are particularly noteworthy as they target fundamental functions of the fusion oncoprotein that drives this disease. The task force will continue to discuss the progress of these and other emerging strategies.
